# The Human Antibody Response to Dengue Virus Infection

**DOI:** 10.3390/v3122374

**Published:** 2011-11-25

**Authors:** Wahala M. P. B. Wahala, Aravinda M. de Silva

**Affiliations:** Department of Microbiology and Immunology, School of Medicine, University of North Carolina, Chapel Hill, NC 27599, USA; Email: wahala@med.unc.edu

**Keywords:** dengue virus, antibody, neutralization, antibody dependent enhancement

## Abstract

Dengue viruses (DENV) are the causative agents of dengue fever (DF) and dengue hemorrhagic fever (DHF). Here we review the current state of knowledge about the human antibody response to dengue and identify important knowledge gaps. A large body of work has demonstrated that antibodies can neutralize or enhance DENV infection. Investigators have mainly used mouse monoclonal antibodies (MAbs) to study interactions between DENV and antibodies. These studies indicate that antibody neutralization of DENVs is a “multi-hit” phenomenon that requires the binding of multiple antibodies to neutralize a virion. The most potently neutralizing mouse MAbs bind to surface exposed epitopes on domain III of the dengue envelope (E) protein. One challenge facing the dengue field now is to extend these studies with mouse MAbs to better understand the human antibody response. The human antibody response is complex as it involves a polyclonal response to primary and secondary infections with 4 different DENV serotypes. Here we review studies conducted with immune sera and MAbs isolated from people exposed to dengue infections. Most dengue-specific antibodies in human immune sera are weakly neutralizing and bind to multiple DENV serotypes. The human antibodies that potently and type specifically neutralize DENV represent a small fraction of the total DENV-specific antibody response. Moreover, these neutralizing antibodies appear to bind to novel epitopes including complex, quaternary epitopes that are only preserved on the intact virion. These studies establish that human and mouse antibodies recognize distinct epitopes on the dengue virion. The leading theory proposed to explain the increased risk of severe disease in secondary cases is antibody dependent enhancement (ADE), which postulates that weakly neutralizing antibodies from the first infection bind to the second serotype and enhance infection of FcγR bearing myeloid cells such as monocytes and macrophages. Here we review results from human, animal and cell culture studies relevant to the ADE hypothesis. By understanding how human antibodies neutralize or enhance DENV, it will be possible to better evaluate existing vaccines and develop the next generation of novel vaccines.

## 1. Introduction

Dengue viruses (DENV) are emerging, mosquito-borne flaviviruses and are the causative agents of dengue fever (DF) and dengue hemorrhagic fever (DHF). Millions of people living in tropical and subtropical regions of the world are infected by DENV each year. Several hundred thousand of these infections progress to DHF, which is a life threatening disease [1]. The DENV complex consists of 4 distinct but related viruses designated as serotypes. DENVs display antibody epitopes that are unique to each serotype and epitopes that are shared between serotypes. People who have recovered from primary DENV infections develop robust antibody responses that cross react with the 4 serotypes. Despite the cross reactivity, antibodies only prevent re-infection by the same serotype (homologous serotype) and individuals are susceptible to a second infection with a different serotype (heterologous serotype) [2,3]. People experiencing a secondary dengue infection with a new serotype face a much greater risk of developing DHF indicating that pre-existing immunity to DENV can exacerbate disease. Antibody dependent enhancement (ADE) of DENV is the most widely supported theory explaining the higher risk of DHF associated with secondary infection [4]. Thus, the antibody response to DENV infection is complex, with potential to benefit or harm the host. Currently many dengue vaccines are under development including live attenuated DENV vaccines, which are entering phase III human trials. An in-depth understanding of the human antibody response to DENV is highly relevant to evaluating vaccines already in the pipeline and for developing new vaccines. Here we review the current state of knowledge about the human antibody response to DENV infection and identify important gaps in our knowledge.

## 2. Dengue Virion Structure

**Figure 1:**
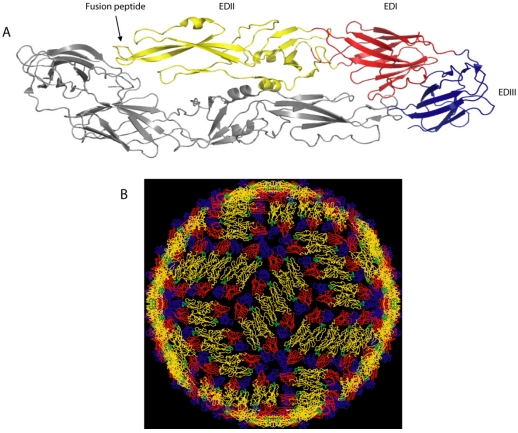
Structure of dengue virus envelope protein (**A**) and the dengue virus particle (**B**). (**A**) E protein on the mature virion is a homodimer and each subunit has three domains designated I (red), II (yellow) and III (blue). (**B**) Arrangement of E proteins on the surface of the virion. Both images A and B are from [12].

The structural arrangement of viral surface proteins plays an important role in dictating how antibodies neutralize viruses. Dengue is an enveloped, positive-strand RNA virus that produces a spherical particle with a diameter of approximately 500A. The viral envelope contains two integral membrane proteins designated envelope (E) and pre membrane (prM). E protein binds to cellular receptors and mediates fusion of viral and cellular membranes during viral entry into cells. E protein is also the main target of neutralizing antibodies. The crystal structures of the E protein of several flaviviruses have been solved [5,6,7,8,9,10]. Individual subunits of E protein consist of three beta-barrel domains designated domains I (EDI), II (EDII) and III (EDIII), with the native protein forming a head-to-tail homodimer (Figure 1). The hydrophobic viral fusion peptide is located at the tip of domain II and is shielded by domain III of the adjacent subunit. Domain III appears to be responsible for binding to cellular receptors as several mutations that affect receptor binding are located in this domain [2]. A detailed picture of how E protein dimers are organized on the surface of the mature, infectious virion has been obtained by combining the crystal structures of E with cryo-EM reconstructions of the entire virion [11] (Figure 1). Each virus particle has 180 monomers of E that are organized into 90 tightly packed dimers that lie flat on the surface of the viral membrane (Figure 1B). Another important feature is that individual E subunits are organized in 2, 3 and 5 fold axes of the T-3 icosahedral structure of the virion. Thus, all the E protein subunits are not in identical environments on the viral surface and steric and other considerations will result in preferential interactions of some E subunits over others with receptors and antibodies [11,12,13].

**Figure 2:**
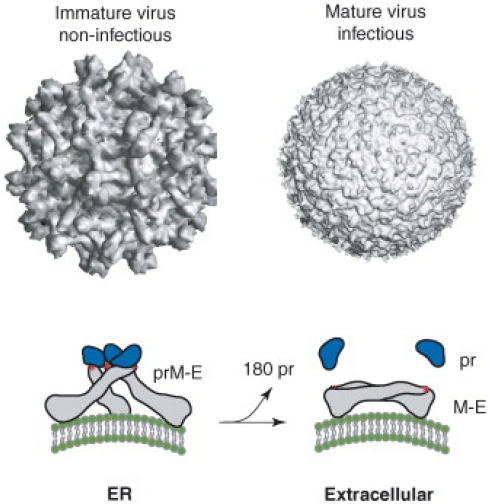
Maturation of dengue virions. Dengue virions bud into the endoplasmic reticulum as immature, non-infectious particles. The surface of immature particles has a jagged appearance because prM and E proteins are initially assembled as trimeric spikes that protrude away from the envelope. In the trans-Golgi compartment a cellular protease cleaves prM protein to generate the mature M protein, which also results in the rearrangement of E protein trimers to form dimers that lie flat on the surface of the envelope creating the smooth surface observed in mature, infectious virions released into the extracellular space. The images reproduced here are from [19].

Dengue virions are assembled on the membrane of the endoplasmic reticulum (ER) and the virus buds into the lumen of the ER as immature virions. Unlike mature virions that have a smooth surface, immature virions that bud into the ER have a rough surface created by trimers of E/prM heterodimers that form spikes on the viral envelope (reviewed in [14]) (Figure 2). These immature particles transition to mature particles during secretion out of infected cells. In the trans-Golgi compartment a cellular protease cleaves prM protein to generate the mature M protein, which also results in the rearrangement of E protein trimers to form dimers that lie flat on the surface of the envelope creating the smooth surface observed in mature, infectious virions (reviewed in [14]) (Figure 2). In practice the process of intracellular DENV maturation appears to be inefficient because many immature and partially mature virions are also released from infected cells [15,16]. Moreover, recent studies indicate that partially mature and even fully immature particles can be infectious under some conditions [17,18]. The structural and maturation properties of DENVs must be considered when defining epitopes engaged by human antibodies and the functional consequences of antibodies binding.

## 3. Studies with Mouse MAbs and the Multi-Hit, Threshold Model of Dengue Neutralization

**Figure 3:**
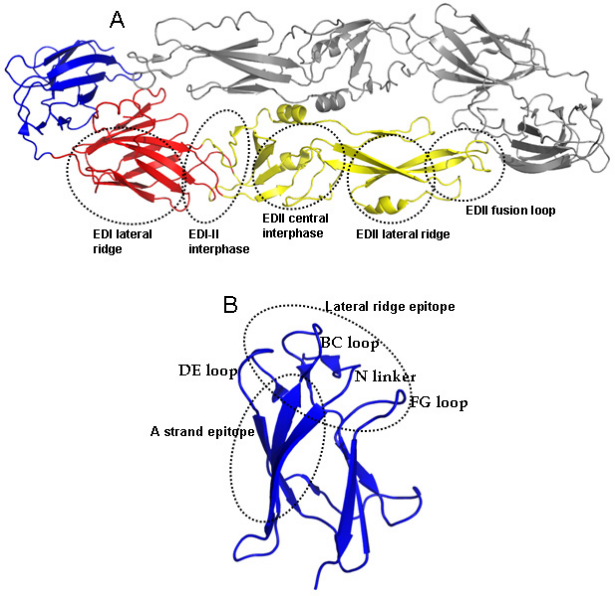
Location of mouse MAb epitopes on DENV E protein. The figure is based on the structure of the ectodomain of DENV3 E protein solved by Modis and colleagues [7]. (**A**) The image depicts the major regions on domains I (red) and II (yellow) recognized by mouse MAbs. (**B**) An enlarged view of domain III (blue) displaying the lateral ridge and A strand epitopes recognized by strongly neutralizing mouse MAbs. Figure originally published in [22].

Most studies to understand how antibodies neutralize or enhance DENV have been done with mouse monoclonal antibodies (MAbs) (reviewed in [20,21]). As E protein is the main antigen exposed on the surface of the virion, mouse MAbs that bind to E protein have been the focus of much study. Although neutralizing mouse MAbs have been mapped to all three domains, the most strongly neutralizing MAbs are serotype-specific and bind to domain III of E protein (EDIII), which protrudes from the surface of the virion (Figure 3). Two partially overlapping epitopes on EDIII designated the lateral ridge and A strand epitopes are the main targets of mouse MAbs that neutralize DENV. The lateral ridge epitope interacts with serotype-specific strongly neutralizing antibodies. Mouse MAbs that bind to the A strand epitope cross react with more than one serotype of DENV and are designated dengue sub complex neutralizing MAbs. 

Mouse antibodies have also been used to explore mechanisms of dengue neutralization. There are 180 potential binding sites on the virion for an antibody that binds to E protein. However, as indicated above all 180 molecules are unlikely to be bound by antibody because of steric effects and limited accessibility of some epitopes. For example, as several EDIIIs are closely packed around the axis of 5 fold symmetry (Figure 1), an antibody binding to one EDIII will obstruct antibody binding to adjacent EDIIIs. It is also becoming clear that the hypothetical structure of the mature flavivirus particle generated by cryo-EM and molecular fitting does not always predict epitope exposure. Binding of some E reactive antibodies depends on the dynamic movement of protein molecules (“breathing”) in the virion particle leading to transient exposure of hidden epitopes. For instance, optimal binding of mouse MAb 1A1D2 to EDIII requires incubation at 37 °C [23]. The structure of the 1A1D2 bound to EDIII indicates that the antibody binds to sites that are transiently exposed during viral “breathing” at 37 °C.

There are at least two if not more distinct mechanisms by which mouse MAbs neutralize DENV in cell culture. Some antibodies neutralize by blocking attachment of the virus to cells, whereas others neutralize by blocking a post attachment step in the entry process (reviewed in [21]). Antibodies that neutralize at a post-attachment step presumably block viral fusion with cellular membranes. Low pH triggered flavivirus fusion requires large conformational changes of E protein molecules and antibodies may interfere with this process required for entry [24,25]. Flavivirus neutralization requires the binding of multiple antibodies (reviewed in [21]). Studies with E16, an EDIII binding MAb that potently neutralizes West Nile virus at a post attachment stage, indicate that ~30 antibodies need to bind for effective neutralization. Based on this and other studies, Pierson and colleagues have proposed that flaviviruses are only neutralized after the number of antibodies bound to the virion exceeds a critical threshold. Important implications of the threshold model are that both the affinity of antibody binding and the total number of accessible epitopes contribute to the neutralization potency of an antibody. Thus, even for an antibody that binds with high affinity, the antibody will fail to neutralize if the number of accessible epitopes is below the threshold required for neutralization. Conversely, a lower affinity antibody may neutralize if many of the epitopes are accessible to binding. The most potently neutralizing antibodies would be those that bind with high affinity to epitopes that are exposed and present in large numbers on the surface of the virion. 

It is unclear if the E16 results can be broadly generalized to most antibodies that neutralize flaviviruses. In other words, is the threshold of 30 true for most if not all flavivirus binding antibodies? Alternatively, does the threshold change depending on the location of binding? Does the threshold model hold for both pre and post attachment neutralizing MAbs? Do the rules depend on the cell type that is being infected as well as the entry receptors and Fc receptors being expressed by the cell? Finally and most importantly, can the threshold model apply to virus neutralization in immune sera when multiple antibodies that bind to different epitopes come into contact with the virion? 

## 4. Human Antibody Response Following Natural DENV Infection

People exposed to DENV infections have detectable specific antibody for decades if not longer. A large fraction of the response cross reacts with all 4 serotypes and even other flaviviruses. In fact the dominance of cross reactive antibodies precludes the use of simple antigen binding assays to identify a flavivirus responsible for infection. The functional, neutralizing antibody response is more specific and useful for identifying the flavivirus responsible for infection. Early studies on the durability of the immune response following DENV infection were conducted by Sabin (1952). Sabin infected naive volunteers with DENV2 (NGC) and DENV 1(Hawaii) and re-challenged these individuals with the homologous virus at different times after the first infection. The subjects were protected up to 18 months (last time point of the study) from re-infection with the same serotype. However, when challenged with a heterologous serotype, cross protection only lasted for 2–3 months after the first infection. Heterologous infection not only produced clinical signs of the disease but also produced sufficient viremia for mosquitoes to acquire infection. Sabin’s work demonstrated that protection was long term against the homologous serotype and only transient against a heterologous serotype. These observations are in agreement with natural history studies of dengue in endemic countries that indicate that primary infections are followed by a several month transient period of broad protection, and a long term protective response that is specific just to the infecting serotype. A recent study by Chan and colleagues provide a molecular basis for this initial broad cross neutralizing response [26]. These investigators demonstrated that early convalescent sera contain high concentrations of weakly neutralizing, cross reactive antibodies capable of forming large virus-antibody aggregates, which then bound to inhibitory FcγRIIB receptors on the surface of monocytes. The protection afforded by this class of antibody is likely to be transient because levels of cross reactive antibodies decline over time. In contrast, potently neutralizing, type-specific antibodies did not require the formation of aggregates for effective neutralization [26]. Type specific neutralizing antibodies can be detected even 60 years after a primary infection [27]. 

Several months after a primary dengue infection, individuals are susceptible to a secondary infection with a new serotype. A hallmark of secondary dengue is a more rapid and elevated antibody response compared to the primary response. The rapid and elevated response is caused by the stimulation of memory B-cells from the primary infection. The first antibodies that appear following a secondary infection neutralize the serotype responsible for the primary infection better than the second virus [28,29]. This phenomenon has been termed “original antigenic sin”, although the molecular basis and mechanisms responsible are incompletely understood [30,31]. Over time, the neutralizing antibody response broadens and a key feature of secondary dengue is a long-lasting response that neutralizes multiple serotypes including serotypes that have not previously infected the individual. Tertiary DENV infections have been documented only rarely, further supporting the notion that secondary infections stimulate long term cross neutralizing antibody that may even be effective against serotypes not encountered previously [32,33]. 

Investigators have also characterized the kinetics and isotypes of the DENV-specific serum antibody response in infected people. Following a primary DENV infection, DENV-specific IgM antibodies appear 4–5 days after onset of fever and are measurable for up to 3 months. IgG antibodies first appear about a week after onset of fever. The IgG response peaks several weeks after infection and then declines to lower levels that persist for decades if not longer. DENV infection mainly induces IgG1 and IgG3 subclasses of antibody indicating a Th1 biased immune response [34,35,36,37]. WNV infection also induces a strong IgG1 response that is protective both in cell culture and in animals [38]. The serum antibody responses are different following primary and secondary DENV infections. Antibodies produced during a second infection arise from naïve B-cells and memory B-cells generated from the primary infection. In secondary infections, the stimulation of B-cell memory leads to a rapid rise in DENV-specific IgG that is measurable even on the first day of symptoms. Moreover DENV‑specific serum IgG titers are much higher in secondary compared to primary infections. For reasons that are not completely understood, in secondary dengue the IgM response is variable, and some cases undetectable. 

## 5. DENV Antigens and Epitopes Recognized by Human Antibodies

In comparison to the work done with mouse MAbs, we are just beginning to learn about the main antigens and epitopes on the dengue virion targeted by human antibodies. Studies have been done with dengue immune sera and human MAbs to define the specificity of the human response. We will start by describing studies with dengue immune sera and then move to studies with human MAbs. After DENV infection, people develop serum antibodies against the structural proteins (E,PrM, C) as well as some of the non-structural proteins (NS1,NS3,NS5) [39,40,41,42,43,44]. Some investigators have reported on higher levels of prM and NS1 antibodies in secondary compared to primary infections [39,40,41]. However, it is unclear if this is due to the overall high levels of antibody in secondary infections or if the antibody responses to prM and NS1 are specifically elevated in secondary infections. Antibodies that bind to NS1 are particularly interesting as this is a non-structural protein that is secreted from infected cells. In one mouse model of lethal dengue infection, NS1 antibodies were protective [45]. It has also been reported NS1 antibodies cross react with self-antigens on platelets and endothelial cells, and investigators have proposed this as a possible mechanism of damage to the vasculature in severe dengue (reviewed in [46]). Although antibodies against NS3 and NS5 have been detected in people, these antibodies are unlikely to influence infection or disease because NS3 and NS5 are intracellular antigens that would not encounter antibody. 

Serum antibodies against DENV E protein have been the focus of several studies as this is the main antigen on the virion surface and the target of neutralizing antibody [39,40,42,43,44,47]. Mutations in the conserved fusion loop on domain II of E protein reduced the binding of immune sera substantially indicating the fusion loop region is a major target of the human antibody response [41]. However, fusion loop antibodies are unlikely to be the target of serotype-specific, potently neutralizing antibody because the fusion loop is well conserved between serotypes. Following up on mouse MAb studies, investigators have tested if EDIII epitopes are the main target of neutralizing antibodies in human immune sera as well. EDIII antibodies are present at low levels in human immune sera and Crill and co-workers observed a correlation between levels of EDIII antibody and neutralization potency of immune serum samples [48]. However, Wahala and Midgely, who used recombinant EDIII protein to deplete sera of EDIII binding antibody observed only a small effect on neutralization following the removal of EDIII binding antibodies [29,49]. Studies with immune sera from horses and people exposed to West Nile virus have also demonstrated that neutralizing antibodies generated following natural infection are not mainly directed to epitopes on EDIII [50,51]. Thus, the main epitopes on DENV targeted by neutralizing antibodies in immune sera remain to be defined. 

## 6. Profiling Antibody Response to Dengue Virus Using Human Monoclonal Antibodies

Several groups have generated panels of dengue reactive human monoclonal antibodies (hMAbs) [17,52,53,54]. The hMAbs were generated by using the method of EBV transformation of memory B‑cells from dengue immune subjects [55]. While it had previously been assumed that E protein was the main target of antibody, the hMAbs indicate a more complex picture. Both E and prM were common targets of human antibody [17,52]. A particularly interesting class of hMAbs, were those that bound to the intact virion but not individual subunits of E or prM [17,52]. The latter class indicates that some B-cell epitopes are only expressed when the viral proteins are assembled to form virions. Studies with dengue immune sera also indicate that only 5–35% of serum antibodies bind to the ectodomain of E expressed as a recombinant antigen, indicating that a substantial fraction of antibodies bind to prM and/or E epitopes that are only expressed on the virion [52].

**Figure 4:**
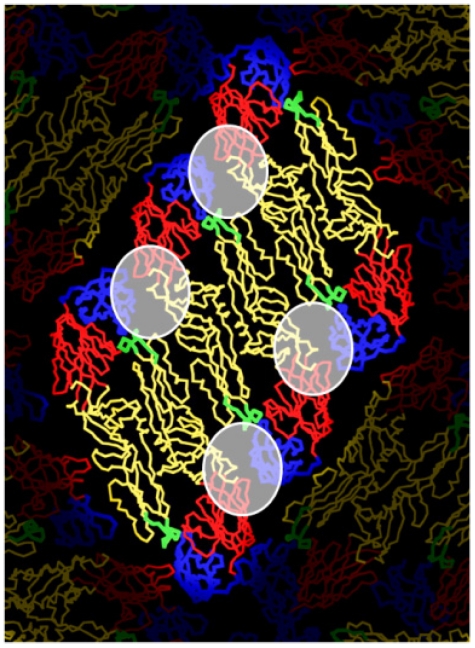
Quaternary structure epitope recognized by West Nile human MAb CR4354. The figure depicts a raft of three E protein homo dimers, which is the basic building block of the flavivirus envelope. The domains of E protein are color coded as described in figure 1. The approximate footprint of human MAb CR4354 is circled. Note that the foot print encompasses EDI/II and EDIII from adjacent dimers. One hundred and twenty CR4354 epitopes are predicted to be available on a virion for antibody binding. Figure adapted from [57,59].

Functionally, the majority of human MAbs produced have been weakly neutralizing and serotype cross reactive, which is consistent with the observation that most dengue specific antibody in circulation is also cross reactive, and weakly neutralizing [17,52,53]. Potently neutralizing hMabs are rare indicating that only small fraction of the total antibody response is responsible for virus neutralization. The neutralizing hMAbs include (1) antibodies that bind to the virion but not recombinant E protein, (2) antibodies that bind to EDI/II, and (3) antibodies that bind to lateral ridge and A strand epitopes on EDIII (see Figure 3) [52]. These results demonstrate that human neutralizing antibodies bind to well defined epitopes on EDIII as well as novel epitopes on EDI/II and epitopes preserved only on the intact virion. Non-human primates also develop potently neutralizing antibodies that bind to novel epitopes. Chimpanzee MAb 5H2 is a potently neutralizing, type-specific antibody that has been mapped to EDI [56]. Of particular interest are studies conducted by Diamond and colleagues with hMAbs generated from people who have recovered from West Nile virus infection [57,58]. Two strongly neutralizing hMAbs bound to the West Nile virion but not recombinant E protein [57,58]. The structure of one of the antibodies (CR4354) bound to the West Nile virion demonstrated that the antibody bound to a complex epitope that spanned EDI/II and EDIII from adjacent dimers (Figure 4), which was consistent with the observation that this class of antibody bound to the virion but not recombinant E protein [57]. These antibodies appear to block infection by cross linking E proteins on the virion and blocking conformational changes required for viral entry and fusion [57]. In summary, these results demonstrate that human antibodies that potently and type specifically neutralize DENV represent a small fraction of the total DENV-specific antibody response. Moreover, these neutralizing antibodies appear to bind to novel epitopes including complex, quaternary epitopes that are only preserved on the intact virion.

### PrM Protein Is a Major Target of the Human Antibody Response

A surprisingly high proportion of hMAbs bound to prM, which is an antigen that has not received much attention as an antibody target [17,52,53]. prM antibodies cross reacted with all 4 serotypes and their neutralization capacity ranged from poor to moderate at best. PrM reactive antibodies enhanced DENV infection in FcγR bearing cells *in vitro*, over a large range of antibody concentration [17,53], indicating their potential involvement in antibody dependent enhancement (ADE) of dengue, which is discussed later in this review. 

## 7. Intra-Serotype Strain Variation and DENV Neutralization

DENVs within each serotype are genetically diverse and classified into distinct genotypes with different geographical distributions and pathogenic potential (reviewed in [60,61]). Despite this genetic variability, it is widely assumed that neutralizing antibody epitopes are conserved among strains belonging to the same serotype. In fact, the current strategy for developing dengue vaccines is based on the assumption that a neutralizing immune response directed to a single strain will protect against most if not all strains of DENV within the serotype. The idea that strain variation within a serotype does not affect neutralization is mainly based on the observation that in human cohort studies one rarely, if ever, observes re-infection with the same serotype. We argue that this observation alone does not mean intra-serotype strain variation is irrelevant for neutralization because most cohort studies have been done in areas where each serotype is represented by the circulating of a single genotype. We need prospective studies that specifically assess what happens when a new genotype is introduced into a population with pre-existing immunity to the serotype. Several recent, laboratory based studied indicate that intra-serotype variation can lead to large differences in antibody neutralization. Blaney and colleagues immunized monkeys with candidate live attenuated dengue vaccines and characterized the immune response in monkeys by using a panel of viruses representing the 4 serotypes and genotypes within each serotype. They observed large differences in neutralization titer when comparing different genotypes of DENV3 [62]. In a study of pediatric dengue cases in Thailand, investigators observed significant differences in the ability of sera to neutralize reference and clinical strains of DENV3 [63]. Guzman and colleagues reported that amino acid sequence differences between DENV3 strains can have strong influences on virus neutralization by murine and human immune sera [64]. Studies with other flaviviruses have also demonstrated that neutralization is dependent on the lineages and strains used in the assay [65,66]. A series of studies from our group and the Diamond group recently demonstrated that the ability of many monoclonal antibodies to neutralize DENV1, 2 and 3 was dependent on the genotype used in the neutralization test [22,67,68,69]. These studies have also led to the identification of point mutations that profoundly alter the neutralization titer. More recently, we have observed a 4 to 10 fold difference in the neutralization titer of human immune sera when recombinant viruses with prM and E from different DENV3 genotypes were used in the neutralization test [70]. Thus, the current paradigm that neutralizing antibody epitopes are conserved within each serotype needs to be tested more vigorously both in the field and in the laboratory.

## 8. *In Vitro versus in Vivo* Neutralization

Studies are needed to assess the relationship between cell culture antibody neutralization of DENVs and *in vivo* protection from infection and disease. Typically cell culture neutralization is based on antibodies binding to the virion and directly interfering with infection. *In vivo* the situation is more complex and antibodies can interact with other components of the immune system such as complement and Fc receptors, which can augment or suppress virus neutralization [4,71,72,73,74,75,76]. Moreover, antibodies can also harness cellular mechanisms such as phagocytosis and antibody dependent cellular cytotoxicity (ADCC) to control DENV infection [77,78]. Despite these differences, some studies have reported on a strong correlation between *in vitro* neutralization potency and *in vivo* protection both with monoclonal antibodies and polyclonal sera [79]. A recent study of infants with maternally derived DENV- specific antibody indicated that an in-vitro neutralization titer of 1:50 is predictive of protection *in vivo* as well [80]. However, some flavivirus antibodies with poor naturalizing activity in cell culture can protect from disease in animal models [45,81,82]. Further studies are needed to define the main mechanisms by which antibodies protect people from severe dengue disease. 

## 9. B-Cell Subsets Involved in the Humoral Response to DENV

As detailed above, most work to date on the human antibody response to DENV has focused on circulating serum antibody and MAb generated from the memory B-cell pool. Studies are needed to identify the actual B-cell subsets activated by dengue and to characterize the functional importance of antibody produced from different B-cell populations. Most antibodies stimulated by viruses are classical T-dependent responses derived from follicular B-cells. The main antibody response to DENV is also likely to involve T-dependent follicular (B-2) B-cells, which differentiate into long lived plasma cells and memory B-cells. Recent studies indicate that less well studied B-cell subsets such as marginal zone B-cells and B1a and B1b cells, which give rise to natural and T-independent antibody responses provide protection from viruses [83]. Studies are needed to assess if similar responses constitute important components of the response to DENV as well. In this regard the recent observation that many human flavivirus antibodies recognize epitopes preserved on the intact virion but not recombinant E protein is intriguing as such antibodies may be produced by the multivalent virus particle directly activating B-cells without any T-cell help [17,52,57]. DENV infection also inhibits type I interferon responses and suppresses antigen presentation by myeloid cells and these effects are likely to influence the quality of the adaptive immune response, including antibody production. We need to invest in human studies and animal models to characterize B-cell subsets involved in the response DENV, with particular attention to how these responses differ in primary *versus* secondary cases, or severe *versus* mild disease cases. 

## 10. Role of Antibodies in Enhancing DENV Infection and Disease

Many studies in different regions of the world have documented that individuals exposed to secondary infections are at greater risk of developing severe disease compared to individuals exposed to primary infections (reviewed in [84]). The leading theory proposed to explain the increased risk of severe disease in secondary cases is antibody dependent enhancement (ADE), which postulates that weakly neutralizing antibodies from the first infection bind to the second serotype and enhance infection of FcγR bearing myeloid cells such as monocytes and macrophages (reviewed in [4]). Here we will discuss evidence for ADE from human, animal and cell culture studies. 

The fact that secondary infections lead to a higher serum viremia and a greater risk of severe disease compared to primary infections strongly suggests that pre-existing immunity (not necessarily antibody) to DENV is a risk factor for severe dengue. The most compelling evidence for ADE has come from studies with infants, who have passively acquired antibodies to DENV from their mothers [85,86,87]. Soon after birth high levels of maternal antibodies appear to protect infants from dengue. Infants born to dengue immune mothers are at greatest risk of developing severe dengue between 6–12 months after birth and this has been attributed to maternal antibody decaying to low, sub-neutralizing levels that enhance DENV. Studies with older children experiencing secondary infections also provide evidence in support of ADE and severe disease. In one study, the ability of immune sera collected just before a second infection to enhance DENV in cell culture was positively correlated with risk of severe disease [88]. However, not all human studies support the ADE theory of DHF/DSS. For instance, in their study with maternally transferred antibodies in infants, Libraty *et al.* [80] did not find a significant association of ADE with the disease outcome. In a prospective study with school children in Thailand, ADE activity of pre-illness undiluted sera did not correlate with the severe disease severity or viral load following secondary infections [89]. While it is difficult to directly compare different studies because of differences in study design and methods, the ADE hypothesis is biologically plausible and supported by sufficient evidence to justify conducting more human cohort studies specifically designed to test the ADE hypothesis.

It has proven challenging to test the ADE hypothesis in animal models because DENV replicates poorly in animals, and DHF and DSS are difficult to reproduce in animals models. Nonetheless a few studies indicate that antibody enhanced infection and severe disease can be reproduced in some animals. Studies with non-human primates [90,91] have demonstrated that animals treated with sub‑neutralizing levels of antibody develop higher serum viremia compared to untreated animals. However, attempts to reproduce DHF in non-human primates have been unsuccessful. Investigators have attempted with some success to develop mouse models of DENV infection and disease (reviewed in [92]). The most successful mouse model of antibody enhanced severe disease is based on infecting interferon receptor-deficient AG129 mice with a mouse adapted strain of DENV2 [93,94]. AG129 mice treated with anti-dengue monoclonal antibodies or polyclonal sera and then infected with DENV developed a lethal vascular leak disease, with similarities to DHF. The cells involved with ADE driven infection were Fc receptor bearing cells including sinusoidal endothelial cells in the liver. In addition to vascular leakage, the disease was characterized by elevated level of cytokines (TNFα, IL-6, and IL‑10), and thrombocytopenia, which is similar to severe DENV illness in humans [93,94]. While specific cellular mechanisms leading to vascular leakage are likely to be different in humans and mice, especially mice deficient in interferon receptors, these animal studies establish that antibodies can enhance viral replication and induce host cytokines responses that lead to clinical outcomes similar to DHF/DSS. Further development of appropriate animal models, including mice reconstituted with human immune cells, is an ongoing and exciting area of dengue research.

The ADE phenomenon can be reproduced in cell culture models. Fcγ receptor bearing human cell lines such as monocytes and macrophages, which are not efficiently infected with DENV alone, become highly permissive to infection in the presence of sub-neutralizing antibody concentrations. ADE has been well demonstrated with monoclonal antibodies and polyclonal sera *in vitro* using Fcγ receptor bearing human cells such as K562, U937, and primary human monocytes, macrophages and dendritic cells. Initially it was believed that ADE simply resulted from a greater number of infected cells producing more infectious virions (extrinsic ADE). However, recent studies by Ubol and colleagues with THP-1 cells (a human acute *monocytic* leukemia *cell* line) indicate that the phenomenon is more complex [95]. DENVs entering THP-1 cells via Fc receptors suppressed type I interferon responses and the activation of cellular antiviral molecules more effectively than DENV infecting the same cells in the absence of antibody [95,96,97]. Moreover, THP1 cells infected in the presence of antibody produced more infectious virus per infected cell compared to cells infected in the absence of antibody. This phenomenon, which has been termed “intrinsic ADE” demonstrates that antibody mediated infection leads to a suppression of the antiviral state within the infected cell and the release of a greater quantity of infectious virions by each infected cell compared to cells infected by antibody-independent entry. Ubol’s observations about intrinsic ADE have been confirmed by other groups working with primary human cells, although the specific mechanisms and molecules implicated appear to depend on the cell type being used or other variables introduced by the different laboratories performing these studies [98,99,100]. With THP-1 cells, antibody dependent infections inhibited type I interferon responses and increased levels of the suppressive cytokine IL-10 [97]. In studies with primary human PBMCs, one group did not observe any differences in type Type I IFN or IL-10 levels [99] whereas another group observed decreased levels of type I interferon and increased levels of IL-6 [100]. Boonak and colleagues compared ADE infections in monocytes, macrophages and dendritic cells and observed increased IL10 production in primary monocytes but not in macrophage or dendritic cells [98]. The picture emerging from these studies is that antibody-complexed dengue viruses infecting cells via Fcγ receptors leads to the suppression of cellular antiviral responses but further studies are needed to better define the cellular pathways and mechanisms that contribute to this phenotype.

## 11. Properties of DENV Enhancing Antibodies

It is well established that almost any dengue specific MAb used at sub-neutralizing concentrations can enhance infection of cultured cells expressing appropriate Fcγ receptors. Studies are needed to identify specific antibody sub-populations in dengue immune human sera that drive ADE in cell culture and animal models and, potentially, in people exposed to secondary infections. When designing these studies, it is important to keep in mind that during a secondary DENV infection, ADE is likely to occur at circulating antibody concentrations present during the acute phase of infection. A recent study demonstrated a quantitative difference in the ability of primary immune sera to enhance a homologous *versus* heterologous DENV serotype [29]. Sera had to be diluted to artificially low concentrations to enhance the homologous virus whereas a heterologous serotype was enhanced at high serum concentrations, likely to be encountered by a virus responsible for a secondary infection. The actual target(s) of antibodies in human immune sera that enhance DENV infection have not been identified. In this regard, the previously mentioned, predominance of prM hMabs has raised the intriguing possibility that these antibodies play a pivotal role in enhancing human DENV infections. DENV produced in cell culture is a complex mix of immature, partially mature and fully mature virions and these preparations are efficiently enhanced *in vitro* and in mice by prM antibodies [101]. By identifying the main antibodies in immune sera with potential for enhancing dengue at physiological concentrations, it will be possible to design vaccines that neutralize without potential for enhancement.

## 12. Summary

Although much remains to be learned about DENV pathogenesis, antibodies have emerged as important host molecules that reduce or exacerbate disease severity. Mouse MAbs have taught us that DENV neutralization is a multi-hit phenomenon requiring the number of antibodies bound to the virus to exceed a critical threshold. More recently, several groups have started to dissect the polyclonal and monoclonal human antibody response to DENV. The human antibody response has features that are similar to and different from the mouse response. The most striking difference is that strongly neutralizing mouse antibodies target epitopes on EDIII, whereas potent human antibodies bind to different epitopes on the viral envelope. Recent studies also indicate that the prM antigen on the DENV envelope is an, hitherto, under-recognized target of the human antibody response, and a potential target of infection and disease enhancing antibody. The field will evolve rapidly as we learn more about the human B-cell response and we develop better animal models for dengue. Over the next few years we are likely to witness many more important discoveries about the interactions of human antibodies with DENV, which will lead to a better understanding of dengue pathogenesis and improved methods for evaluating and developing vaccines.

## Notes Added in Proof

While this manuscript was being reviewed, Mathew and colleagues published a study characterizing human B-cell responses following primary and secondary dengue infection [102]. The investigators demonstrated that people exposed to dengue have B-cells secreting antibodies that bind to E and NS1 proteins and peptides derived from preM protein. Following primary infection, the B-cells produced antibodies that mainly recognized E protein from the homologous serotype. Following secondary infection, the E antibodies were mainly serotype cross-reactive. Antibodies produced after secondary infections displayed avidity differences between serotypes.
